# Isolated Fetal Ascites: A Rare Cause

**DOI:** 10.7759/cureus.8433

**Published:** 2020-06-03

**Authors:** Manikandasamy Veluchamy, Karvendhan Ramasamy, Nishath Liyakat

**Affiliations:** 1 Neonatology, NMC Specialty Hospital, Dubai, ARE; 2 Neonatology, Zulekha Hospital, Dubai, ARE

**Keywords:** isolated fetal ascites, nonimmune hydrops, meconium peritonitis, perforated meckel's diverticulum

## Abstract

A moderately preterm, 2.68 kg, male child was born to para 3 live 3 mother by Cesarean delivery done in view of preterm labor with fetal ascites. The baby had antenatally detected ascites. The baby had distended but soft abdomen. Ultrasound abdomen showed gross ascites. X-ray of the abdomen in supine showed faint lucency in the mid-abdomen region posterior to the bowel gas, which was visualized as free gas along the right half of the abdomen in lateral decubitus position, suggestive of bowel perforation. Laparotomy was done on day three of life, intraoperatively found to have perforated Meckel’s diverticulum. Ascites resolved postoperatively. Isolated fetal ascites is a rare condition but has a favorable prognosis.

## Introduction

Fetal ascites is an abnormal fluid collection in the fetal peritoneal cavity, and it is often the first finding in hydrops fetalis. Ascites is present in 85% of cases of nonimmune hydrops fetalis [1]. Isolated ascites is defined as fluid accumulation in the abdominal cavity without the involvement of fluid accumulation in other body cavities or subcutaneous tissue. It is a rare condition but can be diagnosed easily by ultrasound scanning [2].

Some 30%-75% of cases of isolated fetal ascites resolve spontaneously [3]. The etiology of fetal ascites or associated disorder can be identified in 92% cases, if we follow a systematic protocol for diagnostic workup [4]. The prognosis of isolated fetal ascites depends on the underlying pathology, but generally has been reported to be better than that of hydrops fetalis [5].

Numerous mechanisms have been implicated in the generation of ascites, including abnormal lymphatic drainage, obstruction of venous return due to any space occupying lesion in the thorax, cardiac failure, decreased plasma oncotic pressure seen in fetal anemia, hepatic insufficiency (storage disease) or congenital nephrosis, increased capillary permeability, urinary tract obstruction, and meconium peritonitis. Chromosomal anomalies and intrauterine infections are uncommon causes of fetal ascites [6].

We present a case of prenatally diagnosed isolated fetal ascites secondary to ruptured Meckel’s diverticulum with meconium peritonitis.

## Case presentation

A 25-year-old para 3 live 3 mother was referred to our hospital at 32 weeks gestation with a diagnosis of isolated fetal ascites. This fetal ascites was first observed at 27 weeks ultrasound examination. Further workup revealed no major chromosomal anomalies on noninvasive prenatal testing and a normal fetal echocardiogram. Serology testing for hepatitis B and C, rubella, cytomegalovirus, toxoplasmosis, herpes simplex, and syphilis were negative.

Subsequent ultrasound examinations performed at 28, 30, and 32 weeks of gestation showed an increase in the volume of intraperitoneal ascites without any pleural or pericardial effusion. Fetal abdominal circumference was more than 95th centile with also moderate hydrocele noted. Fetal growth and amniotic fluid volume remained normal on all antenatal ultrasound examinations without any other associated fetal abnormalities.

**Figure 1:**
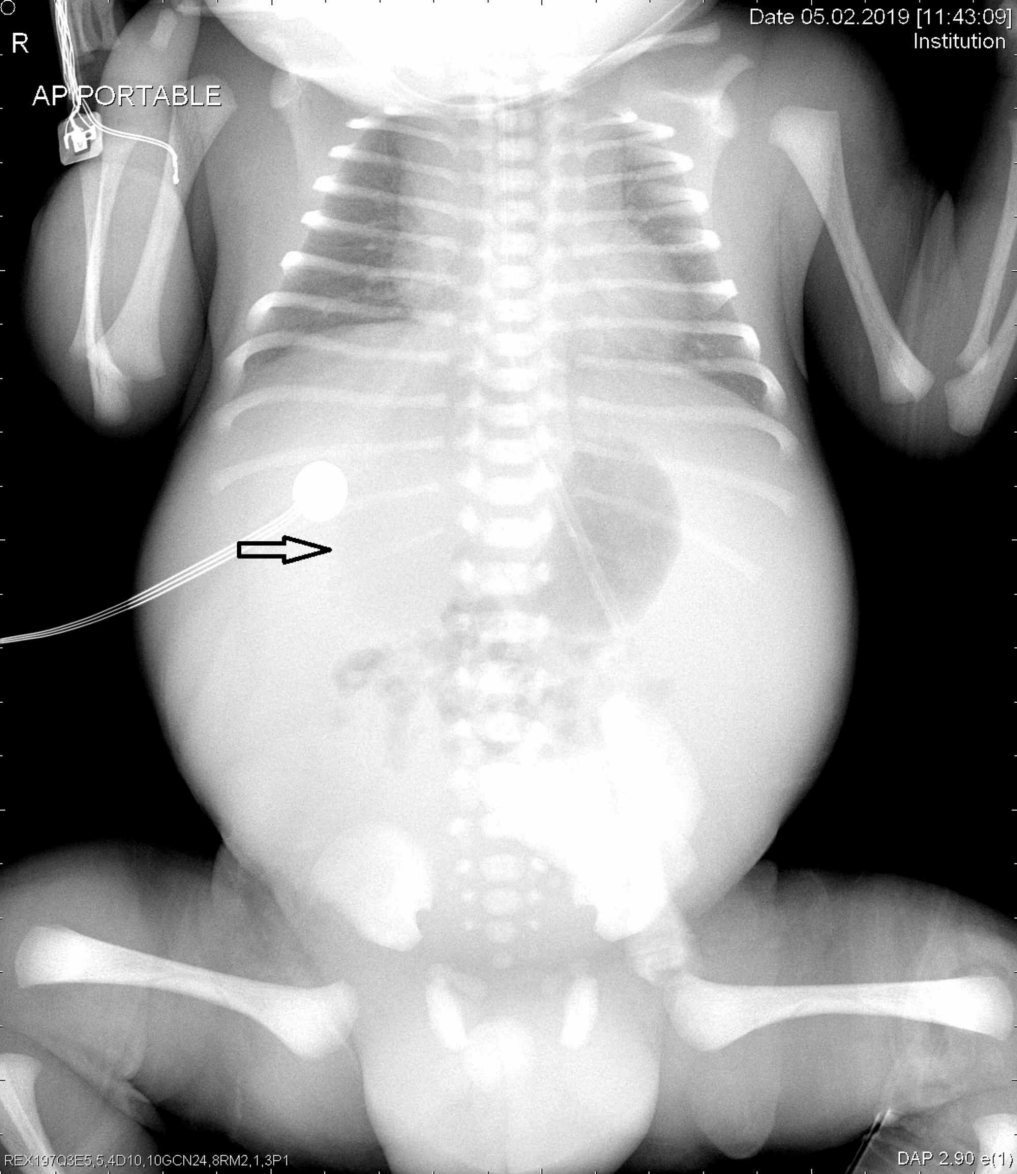
X-ray of abdomen supine. Arrow showing circular faint lucency in the mid abdomen region

**Figure 2:**
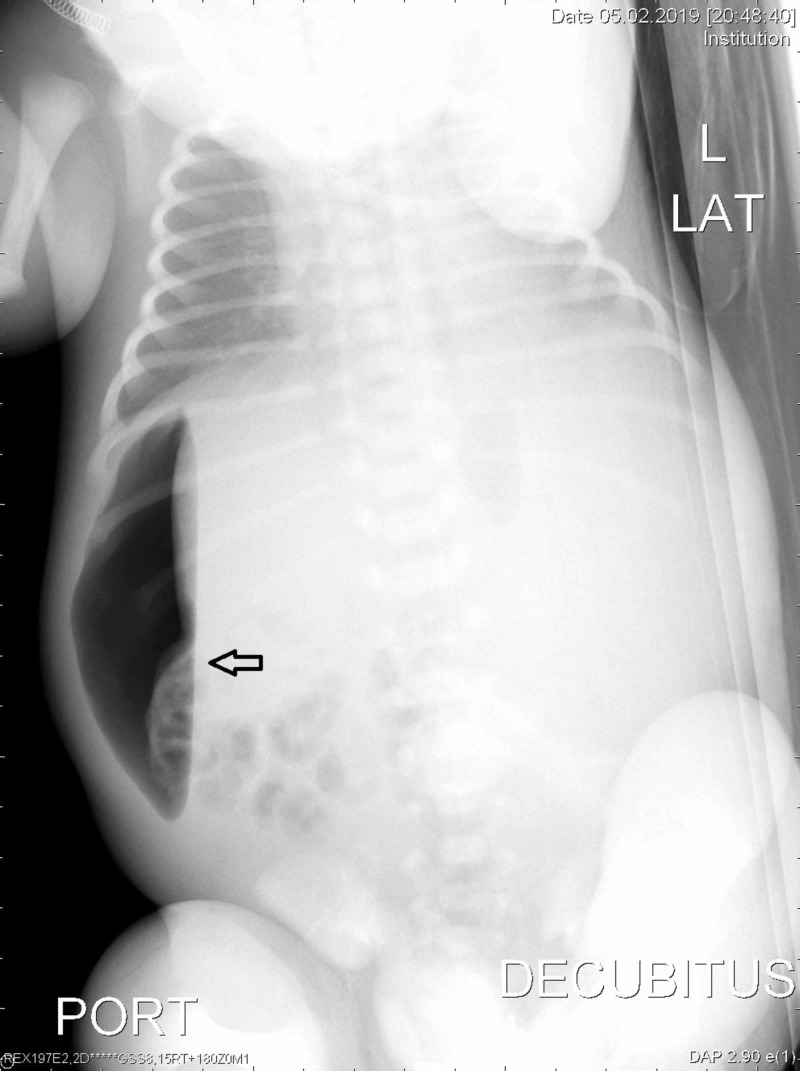
X-ray of abdomen lateral decubitus. Arrow showing free gas along the right half of abdomen

 The patient presented with preterm labor at 32 weeks gestation, and subsequently delivered a male infant by Cesarean section weighing 2680 g with Apgar scores of 3, 7, and 9 at 1, 5, and 10 minutes respectively. The neonate was delivered by a Cesarean delivery in view of nonprogression of labor with fetal ascites. The baby developed respiratory distress soon after birth, but was stabilized on continuous positive airway pressure (CPAP) support in neonatal intensive care unit (NICU). There is no blood group or Rh incompatibility. Further diagnostic evaluation was done in NICU; abdomen X-ray taken in supine position showed faint lucency in the mid-abdomen region posterior to the bowel gas, which is seen as free gas along the right half of abdomen in lateral decubitus position (Figures 1-2). Ultrasound abdomen showed mildly echogenic bowel with subtle increase in cortical echogenicity in both kidneys and gross ascites.

**Figure 3:**
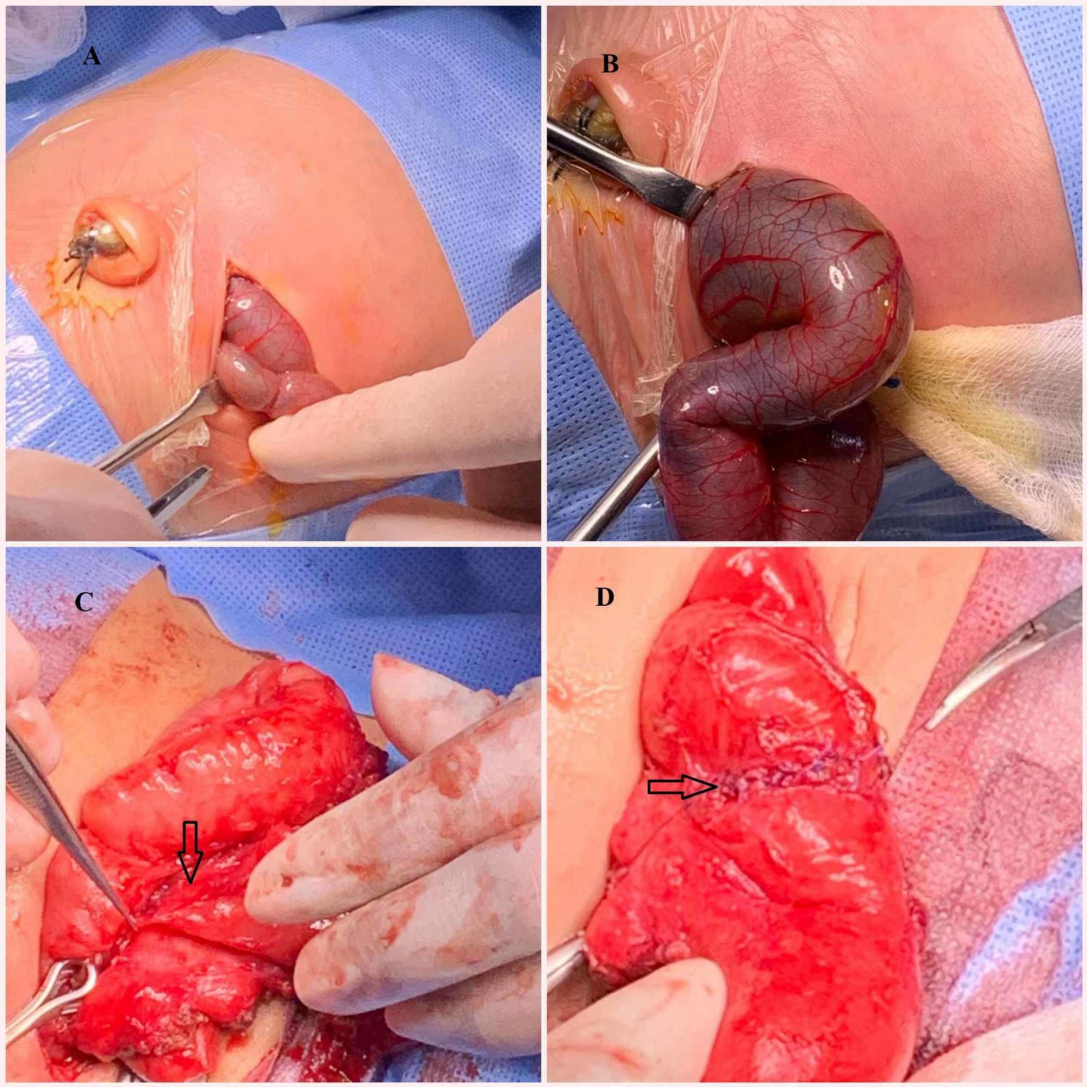
Intraoperative pictures. Intraoperative pictures: A) transverse abdominal laparotomy incision; B) bowel with meconium exteriorized; C) ruptured Meckel's diverticulum resected; D) postresection small bowel sutured.

The neonate had an exploratory laparotomy on day three of life, and was found to have perforated Meckel’s diverticulum with feculent contamination and staining throughout the entire abdomen suggestive of meconium peritonitis. Meckel’s diverticulum was resected and extensive bowel examination was done to rule out the presence of other abnormalities (Figure 3). There is no postoperative complications, gradually feeds were introduced from day six of life, and the baby tolerated feeds well. The baby was discharged from hospital on day 14 of life, without any other problems.

## Discussion

Fetal ascites is diagnosed easily by ultrasound examination. Isolated fetal ascites is diagnosed by the presence of fluid surrounding the liver, spleen, bowel, extra-hepatic portion of the umbilical vein, falciform ligament, or greater omentum. When discovered on an initial sonogram, ascites should be followed by an ultrasound approximately one week later to determine whether progression to fetal hydrops has occurred. When other signs of hydrops like pleural effusion, pericardial effusion, and skin edema are missing, then isolated fetal ascites is assumed. The prognosis and management of isolated fetal ascites is different from ascites associated with hydrops fetalis [2].

 Isolated fetal ascites is most often caused by intra-abdominal disorders due to genitourinary or gastrointestinal conditions; of these obstructive uropathy is the most common cause and 20% are due to gastrointestinal disorders. Meconium peritonitis secondary to bowel obstruction and perforation is the most common gastrointestinal problem causing isolated fetal ascites [6-7].

 A detailed workup using a systematic protocol should be done to find out the etiology. Initial workup should include detailed ultrasound examination to look for the presence of other associated congenital abnormalities. Maternal screening was done for the presence of viral infections such as cytomegalovirus, hepatitis, parvovirus, varicella, herpes simplex, rubella, toxoplasmosis, and syphilis. Maternal screening for isoimmune antibodies, fetomaternal hemorrhage, glucose-6-phosphate deficiency, and thalassemia should also be done. Prenatal genetic testing to look for major chromosomal abnormalities should be done by invasive or noninvasive prenatal testing methods. Invasive prenatal testing methods like amniocentesis and chorionic villus sampling are not only used to identify chromosomal abnormalities but also helpful in identifying fetal infections (TORCH titers) and inherited metabolic diseases. Fetal echocardiogram required to rule out congenital cardiac anomaly or cardiac arrhythmia [8].

 A series of 79 cases of nonimmune fetal ascites was reported by Favre et al. who detected that isolated fetal ascites comparatively has a better survival rate (52%) than ascites associated with other fetal anomalies (42%). They also documented that lower gestational age at diagnosis and the presence of fetal hydrops were associated with lower survival rate [6].

El Bishry reported a series of 12 cases of isolated fetal ascites. Ten out of the 12 cases (80%) survived. Two cases of fetal loss were documented which were diagnosed before 20 weeks of gestation. Two babies out of 10 survived cases required surgery. One baby had ileal atresia and mild hydrocephalus and the other baby had large pseudocyst and bowel atresia, both babies were treated surgically successfully. There was no abnormality detected in eight out of 10 survived babies. Complete resolution of ascites was noted in three cases antenatally and two cases postnatally [4].

 Meconium peritonitis is usually seen secondary to bowel obstruction and perforation. Meconium peritonitis can also be seen in isolation, which usually resolves without sequelae. Various causes of obstruction have been implicated in meconium peritonitis, including meconium ileus, malrotation and midgut volvulus, intussusception, internal bowel hernia, Meckel’s diverticulum, intestinal duplication, vascular insufﬁciency, and small bowel atresia [7].

 However, to our knowledge this is the first report of ruptured Meckel’s diverticulum associated with isolated fetal ascites due to meconium peritonitis.

## Conclusions

Isolated fetal ascites is a rare and separate clinical condition than nonimmune hydrops fetalis. In many cases, fetal isolated ascites may occur as a transient phenomenon, resolving during either the fetal or neonatal periods. Isolated fetal ascites has a favorable prognosis than nonimmune fetal hydrops. A detailed workup should be done to find out the cause, which is important for appropriate treatment and successful outcome.
